# Value and Cost Savings From Access to Multi-disciplinary Rehabilitation Services After Severe Acquired Brain Injury

**DOI:** 10.3389/fpubh.2021.753447

**Published:** 2021-12-01

**Authors:** Laura S. Lorenz, Michael Doonan

**Affiliations:** The Heller School for Social Policy and Management, Brandeis University, Waltham, MA, United States

**Keywords:** health policy, cost-effectiveness, shared decision-making, societal model of health, lifetime savings, traumatic brain injury, stroke, post-acute rehabilitation

## Abstract

Acquired brain injury (ABI) is a major global public health problem and source of disability. A major contributor to disability after severe ABI is limited access to multidisciplinary rehabilitation, despite evidence of sustained functional gains, improved quality of life, increased return-to-work, and reduced need for long-term care. A societal model of value in rehabilitation matches patient and family expectations of outcomes and system expectations of value for money. A policy analysis of seven studies (2009–2019) exploring outcomes and cost-savings from access to multi-disciplinary rehabilitation identified average lifetime savings of $1.50M per person, with costs recouped within 18 months.

**Recommendations:** Increase access to multi-disciplinary rehabilitation following severe ABI; strengthen prevention focus; increase access to case management; support return-to-work; and systematically collect outcome and cost data.

## Introduction

Acquired brain injury (ABI) from traumatic brain injury (TBI), stroke, infectious disease, metabolic disorders, and brain tumors is a major global public health problem (1). A severe ABI (sABI) is any injury to the brain that occurs after birth, disrupts brain function, and has serious consequences (functional, cognitive, psycho-social) for the injured individual. Clinically, severe traumatic brain injury is defined as resulting in loss of consciousness for 6–24 hours or more (2). In the chronic phase of ABI from any cause, lifelong disabilities may affect the ability to work, perform activities of daily living (dressing, paying bills), participate in community life, and/or fulfill a family role. An sABI impacts the life of an individual and their family, and also has a large community, societal and economic toll (3).

The lifetime economic cost of TBIs that occurred in the United States in 2010, including direct medical costs and indirect costs in lost wages, lost productivity, and non-medical expenditures, was estimated to be ~$76.5 billion (in 2010 dollars) (4). Ninety percent of the U.S. economic cost of TBI stems from fatal TBIs and those requiring hospitalization (4, 5). Lifetime costs have increased significantly because advances in emergency medical care and neurosurgery enable more people to survive a hospitalization for brain injury (6, 7). A severe brain injury no longer means an “end” to life for many, but it does mean life changes. Currently, an estimated 47.4% of people who experience a TBI incur lifelong disability in at least one area of function (8, 9). For example, a man hospitalized for a TBI at age 40 could be expected to need assistance with one or more activities of daily living for 23–32 more years (10).

In the U.S. some 20 million Americans are living with disabilities from TBI (from a blow or jolt to the head) and stroke (11, 12). Lack of access to appropriate multidisciplinary post-acute rehabilitation services increases the disability rate, despite evidence that access can increase functional gains (13, 14), quality of life, rates of return-to-work, and savings in long-term care (15). Access to multi-disciplinary rehabilitation can be limited by lack of insurance, coverage limitations, services unavailable close to home, and low understanding of the benefits. Payment through insurance, however, is essential to insuring availability of multi-disciplinary, post-acute rehabilitation services.

**Figure 1:**
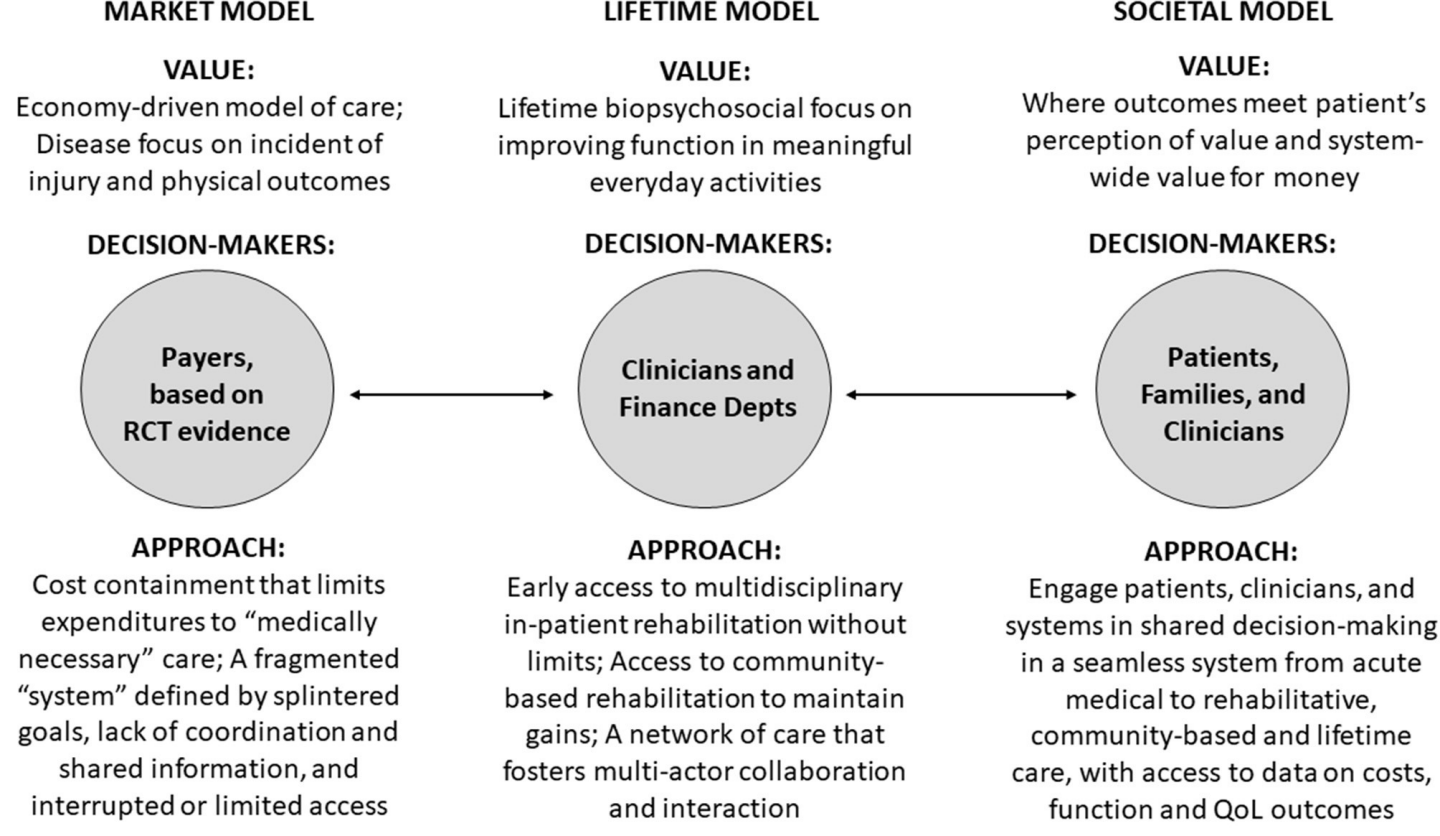
Evolving models of value in access to rehabilitation care for patients with severe acquired brain injury. Source: The Authors. Depts, Departments; QoL, Quality of Life; RCT, Randomized Controlled Trial.

One way to conceptualize value in access to multi-disciplinary rehabilitation after sABI, is to consider three different value models for the provision of healthcare: the market, lifetime, and societal models. The market model informs access to rehabilitation in the market-based health-care system in the U.S. The lifetime model informs healthcare in countries with global healthcare budgets and a universal system of coverage, such as the U.K. (16). The societal model takes into consideration broader population health and wellness goals inside and outside the healthcare system (17). All three models manifest different values, decision-makers, and approaches (see Figure 1).

For the market model value is determined by health payers (17). When payers determine access, rehabilitation care is frequently denied unless the care is “medically necessary” to improve physical function (i.e., walking) and return-to-work (18). Employers are large purchasers of insurance and value low premiums and services that address immediate health needs. Insurance companies have short-term value propositions and are less likely to provide access when health and cost-savings benefits have a long-time horizon and accrue to others. Under the market model, access to rehabilitation services is interrupted or limited and delivered by a fragmented system whose actors have different goals (e.g., free up beds) and little incentive to coordinate across levels (19–21). An example of the market model is the U.S., where different payer systems, including Medicare, Medicaid, Employer-Sponsored Insurance, worker compensation and other options are available—or not available—on a state-by-state basis, and payers play a dominant role in determining access to multi-disciplinary rehabilitation after sABI. In the fragmented U.S. system, worker compensation is the insurance modality most likely to provide access to multi-disciplinary rehabilitation after sABI. The challenge of a compartmentalized system has been noted in the Netherlands, where different entities fund health and rehabilitation care vs. long-term care (22).

For the lifetime model of rehabilitation services, common in countries with integrated, single-payer health systems (16, 23), clinicians determine access based on evidence from research and system data. The lifetime model is concerned with ABI patients' biopsychosocial outcomes and seeks to increase patient independence and participation in life (16, 23). The lifetime model bases decisions about access on clinicians' expert opinions and on rehabilitation savings, projected or actual (16). The goal is to maximize lifelong health benefits across the system in the most cost-effective manner. The lifetime model supports post-acute multi-disciplinary rehabilitation services after an ABI when function can be improved and long-term use of health and social care is reduced, and systematic collection of longitudinal data on services and outcomes to support clinical decision-making (16). Two examples of the lifetime model are Ireland and the U.K., which have integrated, single-payer health systems. In the U.K., regional networks deliver specialist rehabilitation for patients with more complex rehabilitation needs (19). Long-term services and supports are provided by the same single-payer health system. Service utilization and outcomes are tracked longitudinally to inform decision-makers – clinicians – about value and cost-savings vis-à-vis the public investments being made (19).

The societal model considers total societal costs and benefits inside and outside the medical care system. It places a high value on prevention and providing a range of effective services to support independence for individuals. Value in access to multi-disciplinary rehabilitation is determined through shared decision-making by patients, families, and clinicians (17, 21). The societal model relies on a seamless healthcare system from acute care to post-acute rehabilitation to community-based care and coordination with social supports such as vocational training, transportation, and respite care. An example of the societal model is sTBI rehabilitation in Victoria, Australia when the injury occurs due to a transport accident. Victoria's Transport Accident Commission (TAC), established in 1986, is a “no fault” social insurance scheme funded by vehicle registration/insurance fees and returns from investment of unused funds (24). TAC programs fund injury prevention (including road improvement), rehabilitation, case management/coordination of services, income support, return to work, home care, research, and long-term (disability) services and supports for individuals injured in transport accidents (road, train, boat, etc). TAC is notable for engaging youth, patients, families, clinicians, researchers, non-profit organizations, local government entities, and the public in the program (24). TAC services are only available to individuals injured in transport accidents, not for stroke or other acquired brain injuries. There is no comparable sTBI cost-effectiveness study from Victoria to include in our analysis, although the study protocol for an evaluation of the overall injury claims management intervention has been described (25).

In this policy brief, we examine evidence from seven studies in three countries of savings in lifetime care costs from access to multi-disciplinary inpatient rehabilitation supporting physical, cognitive, and social skills after sABI. We provide a model of the continuum of care for sABI and note where policy investments can generate long-term savings. We provide actionable recommendations for policy options at the state and federal level intended to increase access to rehabilitation services after sABI. The analysis uses insights from the lifetime and societal models of healthcare to inform efficiency gains in the market model.

## Policy Analysis

### What Is Known About Rehabilitation Savings After a Brain Injury Requiring Hospitalization

Our policy analysis of seven studies published between 2009 and 2019 explores outcomes and cost-savings from access to rehabilitation services within 12 months of an ABI requiring hospitalization. Study inclusion criteria were (a) published between 2009 and 2019; (b) a TBI or mixed ABI sample of patients with high dependency on admission, which indicated sABI; (c) admitted within 12 months post-injury; and (d) provided access to multi-disciplinary, inpatient rehabilitation services for up to 6 hours per day, 5 or 6 days per week. Study sample size ranged from 33 to 3,289 (median 133). Patient age averaged 42 years (39–49 years). Length of stay in multi-disciplinary inpatient rehabilitation averaged 151 days (89–227 days).

Our calculations of lifetime savings involved several steps. First, we calculated minimum and maximum life expectancy for each sample using a standardized approach for people with TBI (10). Second, for studies that showed savings per week or month we calculated annual savings. Third, to determine lifetime savings, we used (a) an exchange rate approach to transform currencies to dollars and (b) a Purchasing Power Parities (PPP) approach for more accurate inter-country comparison (26) of rehabilitation costs and savings. Fourth, for each study we multiplied annual savings by the minimum and maximum years of life expectancy for each cohort and averaged the two. Finally, we calculated an average savings across all studies by summing their average lifetime savings and dividing by seven.

Across the seven studies lifetime savings from access to multidisciplinary rehabilitation services within 12 months of a severe brain injury averaged $1.58M (SD$.36M) per person using an exchange rate method and $1.50M using a PPP approach (SD$.35M). The cost of services was recouped within 17.2 months (12–27.6 months) on average. Lifetime savings were realized due to patients' increased independence and decreased reliance on services and supports for activities of daily living following discharge (14, 16, 23, 27–30).

The studies calculated rehabilitation savings by comparing the cost of rehabilitation (per person, based on length of stay) and a reduction in post-discharge supervision costs (e.g., care hours) based on reduced dependency or need for supervision (14, 16, 23, 27–30). Typical dependency issues after a severe brain injury include lack of executive function and self-awareness, and increased attention deficit, impulsiveness, disinhibition, irritability, aggression, and mood disorder (14). For all studies, rehabilitation savings were greatest for patients initiating rehabilitation 3–12-months post-injury, though lifetime savings were also noted for patients admitted 2–5-years post-injury (14, 16, 27).

In a U.S. study (30) three independent certified life care planners reviewed anonymized patient reports describing cognitive, communication, mobility, self-care, psychosocial and medical areas at admission and discharge. Each care planner generated a projected cost of care for each patient report. The projected costs from the admission reports were then compared with projected costs from the discharge report file. Savings calculations included costs of long-term care, medical care, equipment, and housing. In Ireland, a study of in-patient rehabilitation cost-effectiveness found that brain-injured patients with greater dependency on admission to rehabilitation achieved the estimated per person cost-savings offset of $56,000 in <16 months (23).

Patient gains in independence were maintained over time. In Great Britain, three intervention studies compared dependency measures at intake, discharge, and 6-months (14, 27, 28). Examples of dependency measures collected at 6-month follow-up were: independence, overnight supervision, part-time supervision, full-time indirect supervision, and full-time direct supervision (27). On average, lifetime savings identified by these U.K. studies ranged from $1.33 to $1.37 million per person. The cost of rehabilitation was recovered within 1–5 years for patients admitted to rehabilitation within 12 months of injury. Calculations used a discounted life expectancy approach for people with brain injury.

A 2019 study in Great Britain (16) used the U.K. Rehabilitation Outcomes Collaborative (UKROC) database to estimate life-time savings in ongoing care after access to tertiary specialist rehabilitation (intensive, in-patient, multi-disciplinary) for brain-injured patients with complex needs. The sample was 3,289 adults (age 16+) with TBI and a length of stay between 8 and 400 days. Mean estimated net lifetime savings averaged $.83 million ($.49M–$1.18M). This study is notable for its sample size, which was 16 times greater than the next largest sample in our analysis.

#### Limitation

A weakness in the studies included in Table 1 is that they do not calculate any decrease in societal costs gained from less reliance on other government programs, improved return-to-work rates, and benefits to families and society through easing of family caregiving and economic burdens. In The Netherlands, researchers conducted a cost analysis of a residential community reintegration program for people injured at least 2 years prior (31, 32). They used Dutch national guidelines to identify the costs of healthcare, informal care, and productivity losses related to participation in the rehabilitation program (31). Societal costs were significantly reduced after participation in the program, and work, education, emotional/behavioral, and independent living outcomes were maintained 3 years later (32). The Netherland studies indicate that initiating access to rehabilitation later than 12-months post-injury also leads to savings. We recommend that future research study societal costs and benefits from access to multi-disciplinary rehabilitation after an sABI.

**Table 1:** Analysis of average, lifetime, per-person rehabilitation cost-savings for patients with sTBI or Mixed sABI (High-Dependency) admitted to multi-disciplinary inpatient rehabilitation <1 Year Post-Injury in Ireland, United Kingdom, and United States (7 studies) (1999–2019).

**Source**	**Sample**	**LOS**	**Measures**	Costs Measured*	Lifetime savings (Exchange)*	Lifetime Savings (PPP)**	Cost Offset (Time to savings)***
Cooney, *Clinical Medicine*, 2016/Ireland	41/mixed sABI/43.5 yrs	93 days	DRS	Direct costs of post-acute rehab care	$1.517M ($2.011M–$1.023M)	$1.310M ($1.737M–$.884M)	15.6 mo
Griesbach, *Journal of Neurotrauma*, 2015/USA	33/sTBI/40.1 yrs	227 days (sTBI)	CIQ CNS DRS LSS MPAI-4 OSS	Projected lifecare costs (pre-rehab vs. post-rehab)	$2.268M ($2.949M–$1.587M)	$2.268M	(not studied)
Oddy, *Brain Injury*, 2013/UK	196/sTBI and stroke/ 41 yrs	183 days	MPAI-4 SRS	Direct costs of post-acute rehab care	$1.502M ($1.940M–$1.064M)	$1.430M ($1.847M–$1.013M)	12 mo
Turner-Stokes, *Brain Injury*, 2007/UK	51/sTBI/39 yrs	183 days	NPDS NPCNA UK FIM+ FAM	Bed-day cost X LOS	$1.926M ($.393M–$2.238M)	$1.835M ($2.329M–$1.341M)	14.2 mo
Turner-Stokes, *BMJ Open*, 2016/UK	190/mixed sABI/46 yrs	103 days	RCSE-M UK FIM+FAM NPDS NPCNA	Episode cost per patient in the rehab unit	$.685M ($.937M–$.432M)	$.652M ($.892M–$.412M)	27.6 mo
Turner-Stokes, *JHTR*, 2019/UK	3,289/sTBI/49 yrs	89 days	UK FIM+FAM NPDS NSPCNA	Cost of rehab	$.833M ($1.176–$.490)	$.793M ($1.120M–$.467M)	15.9 mo
Worthington, *Brain Injury*, 2009/UK	133/mostly sTBI/36 yrs	183 days	ARS FAQ OERS SRS	Direct costs of post-acute rehab care	$2.310M ($2.868M–$1.753M)	$2.200M ($2.731M–$1.669M)	12–24 mo

*Exchange rates: $1.5/£1 (2006–2013); $1.82/€1 (2005 and 2011); **Eurostat, Organization for Economic Cooperation and Development (OECD), Purchasing Power Parities (2012); ***Time point when rehabilitation costs are recouped by rehab savings. Monetary symbols: £= British pound; € = Euro; $ = U.S. dollar. ABI, Acquired Brain Injury; ARS, Accommodation Rating Scale; CIQ, Community Integration Questionnaire; CNS, Center for Neuro Skills Independent Living Scale; DRS, Disability Rating Scale; FAQ, Functional Activities Questionnaire; LOS, Length of Stay; LSS, Living Status Scale; M, million; mo, months; MPAI-4, Mayo-Portland Adaptability Inventory – 4th edition; NPDS, Northwick Park Dependency Scale; NPCNA, Northwick Park Care Needs Assessment; OERS, Occupational Engagement Rating Scale; OSS, Occupational Status Scale; RCSE-M, Rehabilitation Complexity Scale, including medical support; PPP, Purchasing Power Parities; rehab, rehabilitation; SRS, Supervision Rating Scale; sABI, severe acquired brain injury; sTBI, severe traumatic brain injury; TBI, traumatic brain injury; UK FIM+FAM, Functional Independence Measure, version 4 plus a derived Barthel Index; yr, year.

### Where Savings From Investments in Rehabilitation Can Be Realized

**Figure 2:**
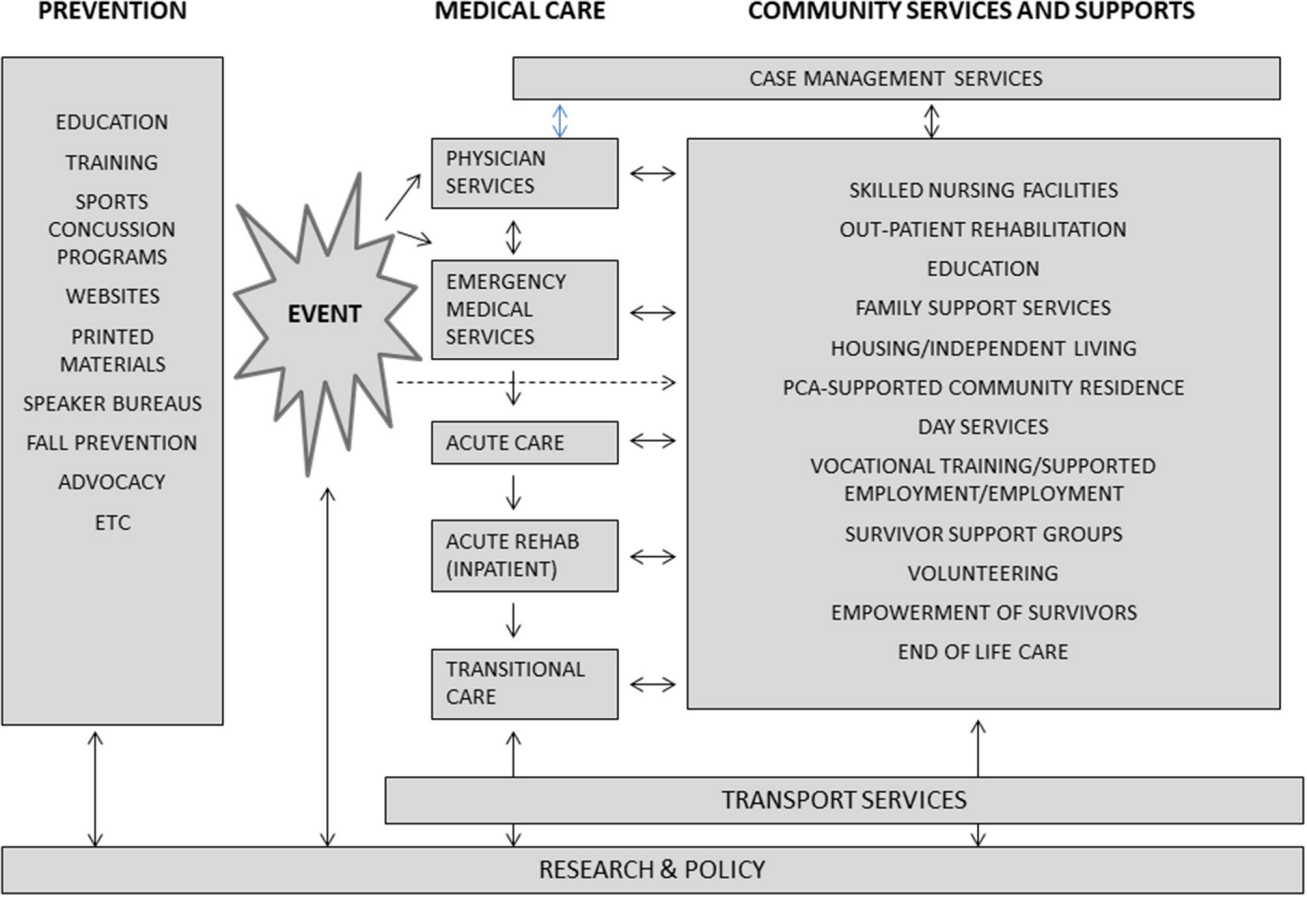
The Continuum of Care for severe acquired brain injury Source: Adapted from the National Association of Head Injury Administrators (33), with permission.

The continuum of care for severe brain injury includes prevention, hospital-based services, post-hospital services, and community-based programs. Traditionally the emphasis has been on acute medical care with less attention on post-acute rehabilitation or community-based care, where most survivor time—and public costs—are spent. Our depiction of the continuum of care for severe brain injury (see Figure 2) illustrates the fluid nature of need as people with severe brain injuries access medical and social care throughout their lives. Figure 2 also illustrates that family support is vital across the continuum to facilitate access to services, support recovery, maintain function, and improve quality of life for people with severe brain injury. The continuum of care provides a context for the services being discussed and our analysis.

In the U.S., the Medical Care phase after a brain injury is largely covered by an individual or a families' health insurance, or by worker compensation if the injury occurred when working. Delays or interruptions in access to rehabilitation services can mean lower gains in function, quality of life, independence, and vocation (34). Within 6 months of their injury, over 30% of U.S. residents who survive sABI lose their private or employer-based health insurance (35). Many with severe injuries incur major debt and are forced into bankruptcy (36). The studies examined demonstrate that improved access to rehabilitation after sABI can lead to savings in medical care at the post-acute care level through reduced length of stay when the care is not interrupted (37, 38) and savings for individuals, families, and society when independence is increased and the need for supervision reduced (39).

## Recommendations

While not explored in this paper, prevention policy to reduce the number of brain injuries would maximize population health and minimize total social costs (4, 40). Enhancing prevention is in keeping with the societal model. Prevention efforts include automobile enhancements to prevent crashes; efforts to reduce distracted, drunken, and drugged driving; sports safety measures to reduce multiple concussions; and fall prevention programs for seniors. One prevention example is Victoria, Australia's efforts to reduce transport accidents through public education campaigns and investments in road infrastructure making high-risk roads and intersections safer for cyclists, pedestrians, and vehicles (41).

The second recommendation is to improve access to rehabilitation after sABI to generate value within the lifetime and societal models of value in healthcare (see Figure 1). The greatest health benefits and savings would accrue from ensuring consistent rehabilitation within the first 12 months of injury. Providing access two or more years post-injury would help to maintain function, maximize independence, and reduce the need for services and supports. One approach to improve access would be to mandate rehabilitation service access nationally as part of mandated coverage requirements under the Affordable Care Act (ACA). Access would then also be required under Medicare and Medicaid, which are the largest providers of services for people with TBI. Alternatively, states could mandate post-acute rehabilitation services through state insurance regulation. Texas has done this since 1995 (42), although the regulation does not apply to all insurance products sold in the state. Large companies that provide health insurance under the Employment Retirement Income Security Act of 1974 (ERISA) for example are exempt from state insurance regulation as is the Medicare program. State action in multiple states, however, would increase the probability of national legislation (43). Implementing these recommendations would adjust the market model to create a level playing field across all insurers and achieve some of the benefits of the societal model.

The third recommendation is universal case management from the time of injury to recovery or end-of-life. It is not enough to have services covered if people do not know what is available and how to access the appropriate care. TBI is, by definition, traumatic causing sudden and massive changes in the lives of individuals and families. Independent case managers provided by state-sanctioned entities not tied to providers or insurers would be provided to all patients regardless of income (24). Case managers would assist patients though the complex medical, economic and social supports necessary to optimize health and independence (44). In addition to medical care, case managers would help provide access to social services such as housing, day programming, and vocational rehabilitation (7, 45, 46), thus increasing the possibility of returning to work, family engagement, community participation, and increased annual earnings (7, 46, 47). Examples of successful case management for people with disabilities from severe brain injury can be found in Missouri, Victoria, Australia, and the U.K. (24, 41, 48). Informing patients, families and providers about the best available care would limit asymmetric information and lead to efficiencies in the market model.

The fourth recommendation is to support back to work efforts. Vocational rehabilitation has been shown to help move people toward greater independence and improved quality of life and save money. Indiana's program “Resource Facilitation,” led to significant cost savings to that state through improved long-term function, reduced annual lost wages, and increased annual earnings (7, 47), identified through research supported by a collaboration of providers, state agencies, advocacy groups, and federal and local funders. Here the societal model informs opportunities for people to engage in communities including the economy.

The final recommendation is to systematically collect service utilization, outcomes, and cost data to better document the costs and savings of rehabilitation services and social supports. The evidence we presented shows unequivocal society and even medical care rates of return from access to rehabilitation after sABI. Having longitudinal data can help to identify where and how savings are achieved and could be maximized, and can help to make an even stronger political case for the upfront investment in multi-disciplinary rehabilitation services after sABI. One approach to data is to create a national brain injury or trauma registry (49), which could be informed by the Traumatic Brain Injury Model System (TBIMS) (50), the OutcomeInfo database (51), and the brain injury registries that already exist in 24 U.S. states. Including demographic and functional measures will help point out where and how optimal health and efficiency goals can be achieved. Longitudinal data are essential for market efficiency.

## Conclusion

Acquired brain injury is a major global public health problem and source of disability. Greater access to multi-disciplinary rehabilitation after a severe ABI will improve lives and save money. Savings are achieved through sustained functional gains, improved quality of life, increased return-to-work, and reduced need for long-term care. The societal and lifetime models of healthcare perceive long-term function of sABI patients as providing value. A societal model centers patient and family needs and promotes public health approaches to prevention and care. Insights from this model can be used to adjust the market model to achieve greater efficiencies and this is reflected in our recommendations. An analysis of seven studies (2009–2019) exploring outcomes and cost-savings from access to rehabilitation after a brain injury requiring hospitalization identified average lifetime savings of $1.50M per person, with rehabilitation costs recouped within 18 months. Our recommendations are to promote prevention, require public and private insurers to provide the range of post-acute rehabilitation services, facilitate access through case management, support back-to-work efforts, and systematically collect and analyze data to better pinpoint where additional health and costs savings can be realized.

The models presented here are informed by work on ethical frameworks to promote health systems change related to access to care for persons with brain injury (21) and on the concept of value in the healthcare system (17). The multi-disciplinary lens' of ethicists, researchers and clinicians in the field of health service research hold promise for evidence-based action that will improve health and save money.

The continuum of care shows the types of care being provided (or not being provided) after a severe acquired brain injury and their typical sequence over an individual's lifetime. Individuals hospitalized for a severe acquired brain injury are not denied acute care, and while saving someone's life can be a heroic act, we argue it is access to multi-disciplinary rehabilitation that can help the individual have better function and quality of life in both the short- and long-term.

## Author Contributions

LSL and MD developed the conceptual framework for the paper. LSL took the lead on the analysis and computations in the piece. MD took the lead on applying the theory to the analysis and drafting the policy recommendations and conclusions. All authors contributed to the article and approved the submitted version.

## Conflict of Interest

The authors declare that the research was conducted in the absence of any commercial or financial relationships that could be construed as a potential conflict of interest.

## Publisher's Note

All claims expressed in this article are solely those of the authors and do not necessarily represent those of their affiliated organizations, or those of the publisher, the editors and the reviewers. Any product that may be evaluated in this article, or claim that may be made by its manufacturer, is not guaranteed or endorsed by the publisher.
